# Prevalence, intensity and endemicity of intestinal schistosomiasis and soil-transmitted helminthiasis and its associated factors among school-aged children in Southern Ethiopia

**DOI:** 10.1038/s41598-022-08333-7

**Published:** 2022-03-17

**Authors:** Zerihun Zerdo, Hilde Bastiaens, Sibyl Anthierens, Fekadu Massebo, Matewos Masne, Gelila Biresaw, Misgun Shewangizaw, Abayneh Tunje, Yilma Chisha, Tsegaye Yohannes, Jean-Pierre Van Geertruyden

**Affiliations:** 1 Department of Medical Laboratory Science, College of Medicine and Health Sciences, Arba Minch University, Arba Minch, Ethiopia; 2 Global Health Institute, Antwerp University, Antwerp, Belgium; 3 Department of Family Medicine and Population Health, Antwerp University, Antwerp, Belgium; 4 Department of Biology, College of Natural Sciences, Arba Minch University, Arba Minch, Ethiopia; 5 Department of Public Health, College of Medicine and Health Sciences, Arba Minch University, Arba Minch, Ethiopia

**Keywords:** Diseases, Risk factors

## Abstract

Preventive chemotherapy (PC), the main strategy recommended by the World Health Organization to eliminate soil-transmitted helminthiasis (STH) and schistosomiasis (SCH), should be strengthened through identification of the remaining SCH transmission foci and evaluating its impact to get a lesson. This study was aimed to assess the prevalence of STH/SCH infections, the intensity of infections, and factors associated with STH infection among school-aged children (SAC) in Uba Debretsehay and Dara Mallo districts (previously not known to be endemic for SCH) in southern Ethiopia, October to December 2019. Structured interview questionnaire was used to collect household data, anthropometric measurements were taken and stool samples collected from 2079 children were diagnosed using the Kato-Katz technique. Generalize mixed-effects logistic regression models were used to assess the association of STH infections with potential predictors. A P-value less than 0.05 was considered statistically significant. The prevalence of *Schistosoma mansoni* in the Dara Mallo district was 34.3% (95%CI 30.9–37.9%). Light, moderate, and heavy *S. mansoni* infections were 15.2%, 10.9%, and 8.2% respectively. The overall prevalence of any STH infection was 33.2% with a 95% confidence interval (CI) of 31.1–35.3%. The intensity of infections was light (20.9%, 11.3% & 5.3%), moderate (1.1%, 0.1% & 0.4%) and heavy (0.3%, 0% & 0%) for hookworm, whipworm and roundworms respectively. The overall moderate-to-heavy intensity of infection among the total diagnosed children was 2% (41/2079). STH infection was higher among male SAC with Adjusted Odds Ratio (AOR) of 1.7 (95%CI 1.4–2.1); occupation of the household head other than farmer or housewife (AOR = 0.5; 95%CI 0.3–0.8), middle [AOR = 1.1; 95%CI 1.0–1.3] or high [AOR = 0.7; 95%CI 0.5–0.9] socioeconomic status. Dara Mallo district was moderate endemic for *S. mansoni*; and it needs sub-district level mapping and initiating a deworming campaign. Both districts remained moderate endemic for STH. Evidence-based strategies supplementing existing interventions with the main focus of the identified factors is important to realize the set targets.

## Introduction

Schistosomiasis (SCH) and soil-transmitted helminthiasis (STH) are the two most common Neglected Tropical Diseases (NTDs) affecting economically deprived people living in warm and moist tropical areas^1^. SCH is caused by blood flukes of the genus Schistosoma. Humans become infected by these flukes when the larval form of the parasite, released into the water by the freshwater snail host, penetrates the skin during contact with infested water. It was reported from 78 countries, but 90% of moderate-to-heavy infections requiring preventive chemotherapy (PC) live in Africa. There are two major forms of SCH (intestinal and urogenital) with the intestinal form caused by *S. mansoni* being the most common in Ethiopia^2^.

STH is caused by helminths of the roundworms (*Ascaris lumbricoides*), the whipworms (*Trichuris trichiura*), and the hookworms (*Ancylostoma duodenal* or *Necator americanus*). Humans are infected by these helminths through ingestion of food or water contaminated with embryonated eggs of roundworms and whipworms or active penetration of the skin by filariform (infective 3rd stage) larvae of hookworms from infested soil^1^. The World Health Organization (WHO) update on March 2020 indicated that about 1.5 billion or 24% of the world population was infected by STHs^3^.

The morbidities associated with SCH were due to the reaction of the body to the eggs lodged in the blood vessels. Intestinal SCH can result in abdominal pain, diarrhea, and bloody stool. In the advanced stage of the disease, there is an enlargement of the spleen and liver, which is associated with accumulation of the fluid in the peritoneal cavity and hypertension of abdominal blood vessels. In children, SCH causes anemia, stunting, and reduced ability to learn. In some chronic cases, SCH can lead to death. The WHO estimated that the annual death rate due to SCH was 200,000 globally^2^. The symptoms of STH depend on the intensity of infection and are attributable to their chronic nature and insidious impact on the health and quality of life than mortality. In moderate-to-heavily infected individuals, STH impairs the physical growth, cognitive development, and micronutrient deficiencies leading to poor school performance and absenteeism in SAC; reduced work productivity and adverse pregnancy outcomes in adults^4^.

In Ethiopia, about 79 million people, including 25.3 million SAC were living in STH endemic areas while the corresponding numbers for intestinal SCH were 37.3 million and 12.3 million in 2013^5^. The 2013 and 2015 national SCH and STH mapping carried out in Ethiopia revealed that the overall prevalence of STH and SCH infections was 21.7% and 3.5% respectively^6^. Ethiopia launched the national NTD control program in November 2015 and targeted to eliminate morbidity due to STH and SCH among SAC by 2020 and break transmission by 2025. In its 1st national PC campaign, Ethiopia achieved 100% geographic coverage and the individual treatment coverage was 77% and 76.5% for SCH and STH respectively^5,7^. However, the coverage validation survey undertaken in March 2019, indicated that the treatment coverage varied widely per district ranging from 54.4% to 91.6% for STH and 59.7% to 89.6% for SCH^8,9^.

To overcome the morbidities from STH and SCH, the WHO recommends chemoprevention as a public health intervention to reduce the worm burden for preschool children, SAC, adolescent girls, women of the reproductive age group, and adults in certain high-risk occupations^1^. Though PC and other supplementary interventions reduced the prevalence and intensity of STH infections, the gain was spatiotemporally heterogeneous, suggesting stringent monitoring and evaluation of the deworming process to be undertaken in different contexts^10^.

SCH and STH morbidity elimination and transmission breaking targets need consistent monitoring and evaluation of the interventions to get lessons and customize the existing interventions. However, the SCH transmission foci are not exhaustively identified, the impact of the existing interventions on the distribution of STH and SCH were poorly addressed in hard-to-reach areas like Dara Mallo and Uba Debretsehay districts. Therefore, the present study was primarily aimed to estimate the prevalence of STH and SCH infections, the intensity of the infections after 5 years PC against STH among SAC. In addition, factors affecting STH infections were assessed in Dara Mallo and Uba Debretsehay districts. This study was embedded in baseline data collected for a cluster-randomized trial that is used to assess the effect of malaria prevention education on malaria, anemia, and cognitive performance of SAC in the study area. The trail was registered in PACTR with Trail registration identification of PACTR202001837195738 on 21 January 2020. The findings of this cross-sectional study will be used by decision-makers to customize the existing interventions or look for supplementary interventions to accelerate reaching the set targets of morbidity elimination and transmission break of STH and SCH.

## Methods

### Study setting

**Figure 1:**
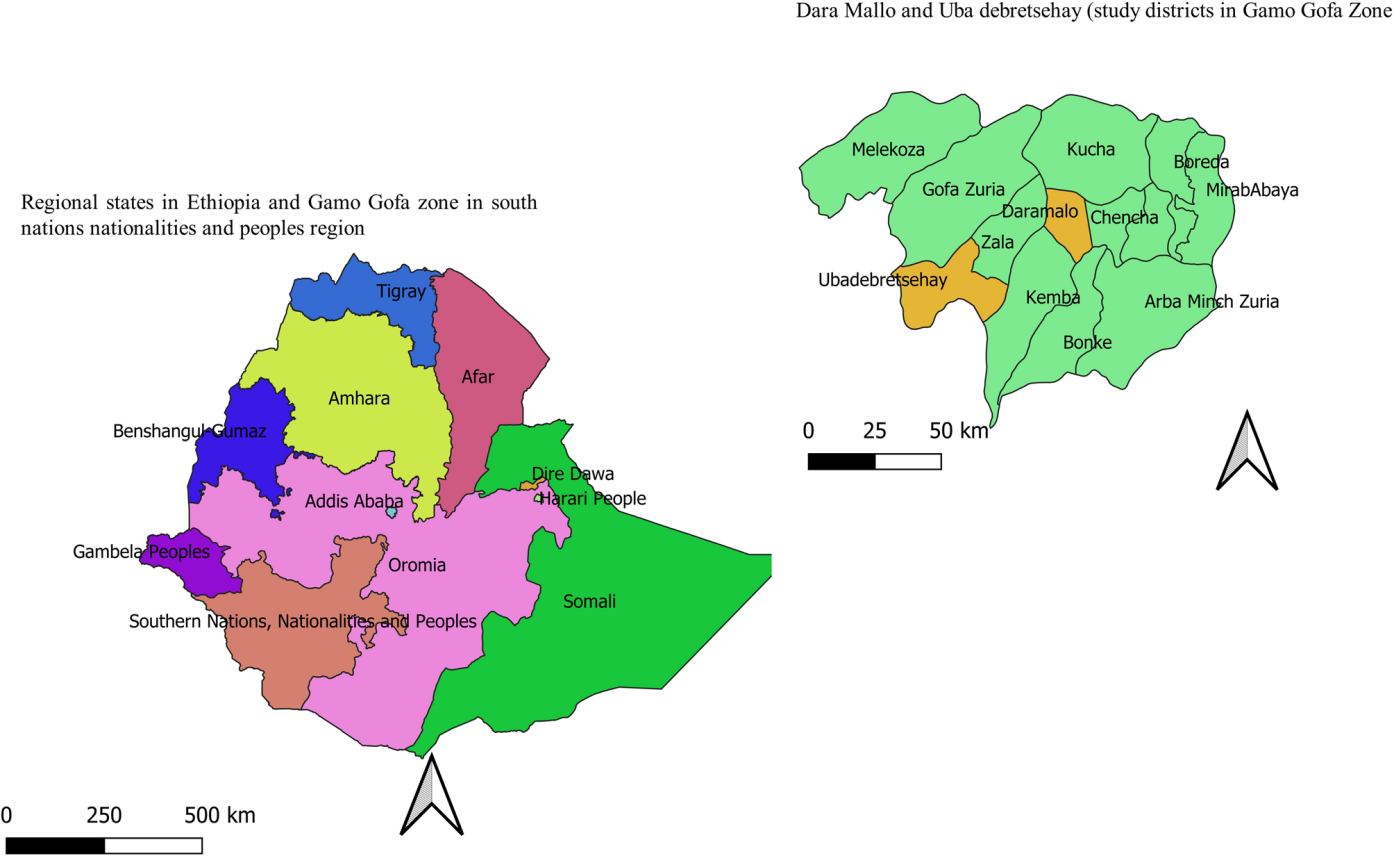
Study districts from former Gamo Gofa zone, Southern Nations, Nationalities and peoples region, Ethiopia.

This study was conducted in Dara Mallo and Uba Debretsehay districts in Gamo and Gofa Zones respectively. Both zones are found in Southern Nations, Nationalities, and Peoples Regional (SNNPR) state which is the 3rd populous region among the 9 regional states in Ethiopia. Both districts are located in the western part of Arba Minch town, capital of the former Gamo Goffa zone, as indicated in Fig. 1. Based on the 2007 national census, a total of 150,145 people were living in the two districts and of whom 76,550 (51%) were male^11^. According to the recent update of the population by the respective districts, there were a total of 94,396 people in the Uba Debretsehay district and 110,207 people in the Dara Mallo district. All 32 schools (20 from Uba Debretsehay and 12 from Dara Mallo) involved in the study are located in an altitude range below 2000 MASL.

### Study design and sample size

A cross-sectional study was undertaken by recruiting children aged 5–14 years, attending their education in grades 1, 2, and 3 in the 32 primary schools in the study area. The sample size to address the primary objective of this study was computed by using single population proportion formula by assuming that 50% of the children were infected by either STHs or intestinal schistosomiasis (P); 5% margin of error (d) and design effect of 3.0

$${\text{N}} = \, \left( {{\text{Z}}\upalpha /{2}} \right){2}*{\text{P}}\left( {{1} - {\text{P}}} \right)/{\text{d2}}$$

After adding the 10% non-response rate, the estimated sample size was 1268. However, this study was embedded in a large cluster-randomized trial conducted to evaluate the effect of malaria prevention education on malaria, anemia, and cognitive development among SAC in the study area. The estimated sample size of the trial was 2304. Since increasing sample size reduces the sampling error and increases the power of the study, we have included all children who consented, participated in the interview, and gave stool specimens for the laboratory diagnosis.

### Sampling techniques and method of data collection

Seventy-two children from each school were selected using a systematic random sampling technique with a class roster as the sampling frame. The number of participants from each grade level (grade 1 to 3) was determined by the relative contribution of students to the total enrolment in each school. Children who were aged between 5 and 14 years and attending their education in the schools during the data collection period were included in the study while those mentally not fit to respond to questions directed to them, with physical problems to measure their height were excluded from the study. Children enrolled in the study were used to trace their households. Participants' households were approached by trained data collectors for interview and observation. A pretested and structured questionnaire, uploaded to the android version Open Data Kit (ODK) application, was used to collect data through face-to-face interviews with mothers/caretakers of the selected children on socio-demographic characteristics, socioeconomic factors, and water access. It was also used to observe the type of sanitation facilities and their cleanliness and a face-to-face interview with the selected SAC on hygienic practice. The coordinates of household location were taken using GPS (Global Positioning System). The data collectors were trained on how to use the ODK for data collection and ethical procedures.

### Anthropometric measurements

Anthropometric measurements were taken according to the WHO guideline^12^. Children were weighed by using Seca digital scales. The scales were placed on a hard and flat surface. Children were weighed with their lightweight clothing (excluding shoes, belts, socks, watches, and jackets). Each child's weight was measured to the nearest 0.1 kg by two individuals. If the two measurements differ by more than 0.1 kg a third measurement was made and the two closest measurements with a difference less than or equal to 0.1 kg were recorded.

When measuring the height of a child, he or she stood with his/her back against the board, his/her heels, buttocks, shoulders, and head touching a flat upright sliding headpiece. The child's legs were placed together with the knees and ankles brought together. Children were asked to take in a deep breath. The child's height measurement was taken when the child had maximum inspiration. The headpiece was brought down onto the uppermost point on the head and the height was recorded to the nearest 0.1 cm at the examiner's eye level. In this case, also, the height was measured by two data collectors, and a difference of 0.5 cm was tolerated. In cases when the difference between the two measurements was greater than 0.5 cm, a third measurement was taken and the nearest two measurements were recorded like the measurement done for weight.

### Stool examination

After instructing children on how to collect stool specimens without contamination with soil, urine or any dirty material, a polyethylene screw cupped stool container was given to selected students to bring the stool specimen. The collected stool specimen was transported to Wacha and Beto health centers for laboratory diagnosis within the same day of its collection. Single stool smear was prepared following the manufacturer's instruction of the Kato-Katz thick smear technique. The prepared stool is left for 30 min after preparation and examined under a bright-field microscope within an hour of smear preparation. The template used for the preparation of the smear was 41.7 mg^13^. The number of helminth eggs present in the whole smear was counted and written in laboratory report form designed for this purpose^14^.

### Data analysis

The number of egg counts in the whole preparation of Kato-Katz thick smear and data collected using android version ODK application was converted to comma-separated values (CSV) file. Multiple factor analysis by using household assets, housing conditions, source of drinking water, agricultural land area, and the number of domestic animals was used to generate the wealth index of a household to measure socioeconomic status. The 1st dimension was categorized into tertiles to classify the household economic status into poor, middle, and high. These data were subjected to descriptive statistical analysis like the proportion for categorical variables and plots and descriptive measures for continuous data.

The total number of ova counted from the whole preparation of the Kato-Katz thick smear was multiplied by 24 to get the number of ova per gram of stool specimen. This count was used for further classification of the infections into light, moderate and heavy infections. The prevalence of STH infection in each school involved in the study was computed to determine the endemicity of the infection: low, moderate, or high endemic^13^.

The GPS coordinates taken from each household point were used to calculate the average point to represent the geographic location of the respective schools. Geospatial distribution of endemicity of the infections per school was presented by using quantum geographic information system (QGIS) version 3.12.1. This software is used to create map of the study area and the endemicity of STH and *S. mansoni* infection per schools studied^15^.

Univariable and multivariable mixed-effects logistic regression models were used to assess the association between STH infections with the potential predictor variables by taking schools as a random variable. Selection of variables for multivariable mixed-effect logistic regression was made through stepwise backward variable selection method in which variable with the largest P-value is removed from the model and checked for AIC. If removal of a variable from the model improves the AIC value, it is removed from the model, otherwise, it is maintained in the model. In all cases, P-values less than 0.05 were considered statistically significant.

### Ethical approval and consent to participate

The trial to which this study was imbedded was approved by the written consent procedure to be followed by the Institutional Research Ethics Review Board (IRB) in College of Medicine and Health Sciences at Arba Minch University with the reference number of IRB/154/12. The trial was conducted according to the Consort 2010 statement: extension to cluster randomized trials^16^. It is registered in the Pan African Clinical Trials Registry with a trial ID of PACTR202001837195738. Official request of the permission letter to conduct the research was submitted to district health offices and education offices in Dara Mallo and Uba Debretsehay districts. Support letter written by the respective education offices to support and participate in the study was given to each of the selected schools. The school headmasters were given a support letter from the education offices and written consent was obtained. Parents of the selected SAC were requested to come to the schools and written informed consent was obtained before data collection. Written informed consent was obtained from each of the selected student's parents at the school and documented in the college of medicine and Health Sciences in Arba Minch University. Children positive in stool examination were treated by using a single dose of 400 mg Albendazole for STH and 40 mg of Praziquantel /kg in two divided doses for *S. mansoni*.

## Results

A total of 2304 SAC were approached to participate in the study, but complete data were obtained from 2079 participants (1362 from Uba Debretsehay district), giving a response rate of 90.2%. Of the total participated children, almost half (1040/2079) were male and the mean age of SAC was 8.7 years with a standard deviation (SD) of 1.5 years. The detailed socio-demographic characteristics of participants were indicated in Table 1.

**Table 1:** Socio-demographic characteristics of participants involved in the study per districts (Uba Debretsehay or Dara Mallo), Southern Ethiopia, 2019.

Variable	Categories	N (%)	Dara Mallo	Uba Debretsehay
			N (%)	N (%)
Place of residence	Rural	1779 (85.6)	618 (86.2)	1161 (85.2)
	Semi-urban	157 (7.6)	87 (12.1)	70 (5.1)
	Urban	143 (6.9)	12 (1.7)	131 (9.6)
Gender of the household head	Male	1930 (92.8)	677 (94.4)	1253 (92.0)
	Female	149 (7.2)	40 (5.6)	109 (8.0)
Age of the household head	≤ 34	514 (24.7)	181 (25.2)	333 (24.4)
	35–49	1399 (67.3)	459 (64.0)	940 (69.0)
	≥ 50	166 (8.0)	77 (10.7)	89 (6.5)
Occupation of the household head	Farmer	1707 (82.1)	559 (78.0)	1148 (84.3)
	Civil servant	145 (7.0)	75 (10.5)	70 (5.1)
	Merchant	107 (5.1)	48 (6.7)	59 (4.3)
	Housewife	70 (3.4)	13 (1.8)	57 (4.2)
	Others	50 (2.4)	22 (3.1)	28 (2.1)
Educational status of household head	Illiterate	1153 (55.5)	247 (34.4)	906 (66.5)
	Literate	926 (44.5)	470 (65.6)	456 (33.5)
Occupation of the child mother/caretaker	Housewife	1735 (83.5)	583 (81.3)	1152 (84.6)
	Farmer	164 (7.9)	44 (6.1)	120 (8.8)
	Civil servant	75 (3.6)	44 (6.1)	31 (2.3)
	Merchant	81 (3.9)	36 (5.0)	45 (3.3)
	Others	24 (1.2)	10 (1.4)	14 (1.0)
Educational status of child mother/caretaker	Illiterate	1509 (72.6)	412 (57.5)	1097 (80.5)
	Literate	570 (27.4)	305 (42.5)	265 (19.5)

### Prevalence, intensity and endemicity of infections

Overall, the prevalence of intestinal helminth infection was 44.6% (95%CI 42.5–46.8%) and infection only by STH was 33.2% (95%CI 31.1–35.3%). The prevalence of *S. mansoni* among SAC in the Dara Mallo district was 34.3% (246/717) with 95%CI of 30.9–37.9%. The prevalence of each species of parasite, intensity of the infection and the median (IQR) of the egg count among infected children was presented in Table 2.

**Table 2:** Prevalence, intensity of infection and the median (IQR) of egg count among SAC in Dara Mallo and Uba Debretsehay districts, southern Ethiopia, 2019.

Parasite	Prevalence	Percentage of intensity of infection			Median (IQR)
		Light	Moderate	Heavy	
Hookworms	22.2% (n = 465)	20.9	1.1	0.3	120 (48–288)
Whipworms	11.4% (n = 237)	11.3	0.1	0	48 (24–72)
*Roundworms*	5.7% (n = 119)	5.3	0.4	0	168 (48–1260)
*S. mansoni*	34.3% (n = 246)	15.2	10.9	8.2	144 (48–378)

**Figure 2:**
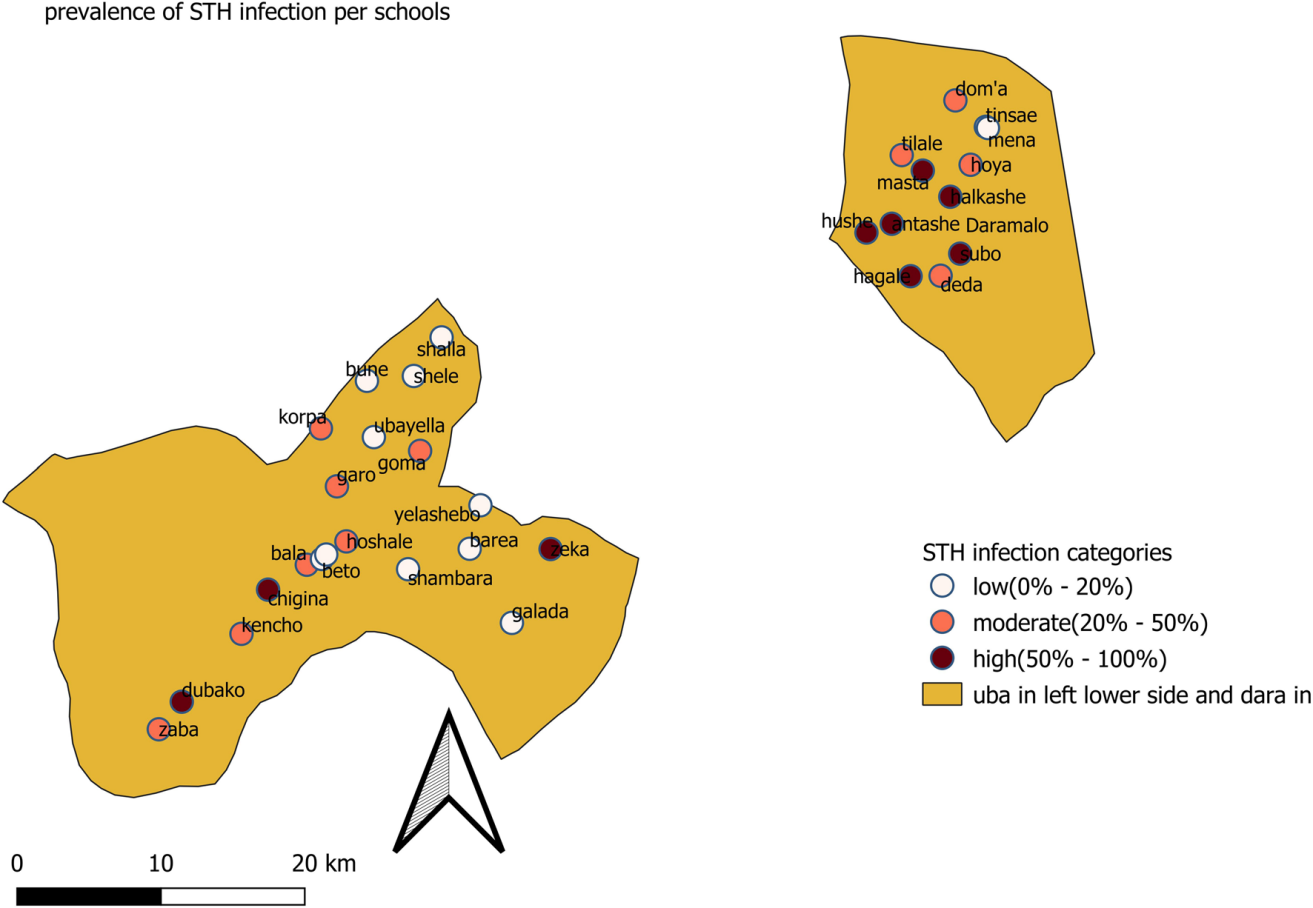
STH infection endemicity per schools in study districts in Southern Ethiopia, 2019.

The prevalence of STH infection varied widely per school. Of the total 32 schools involved in this study, in 10, 13, and 9 of the schools, the prevalence was low, moderate, and high endemic respectively. Half of the schools (n = 6/12) involved in the study from the Dara Mallow district were high endemic for STH. The geospatial distribution of low, moderate, and high prevalence of infections per school in each district was depicted (Fig. 2).

**Figure 3:**
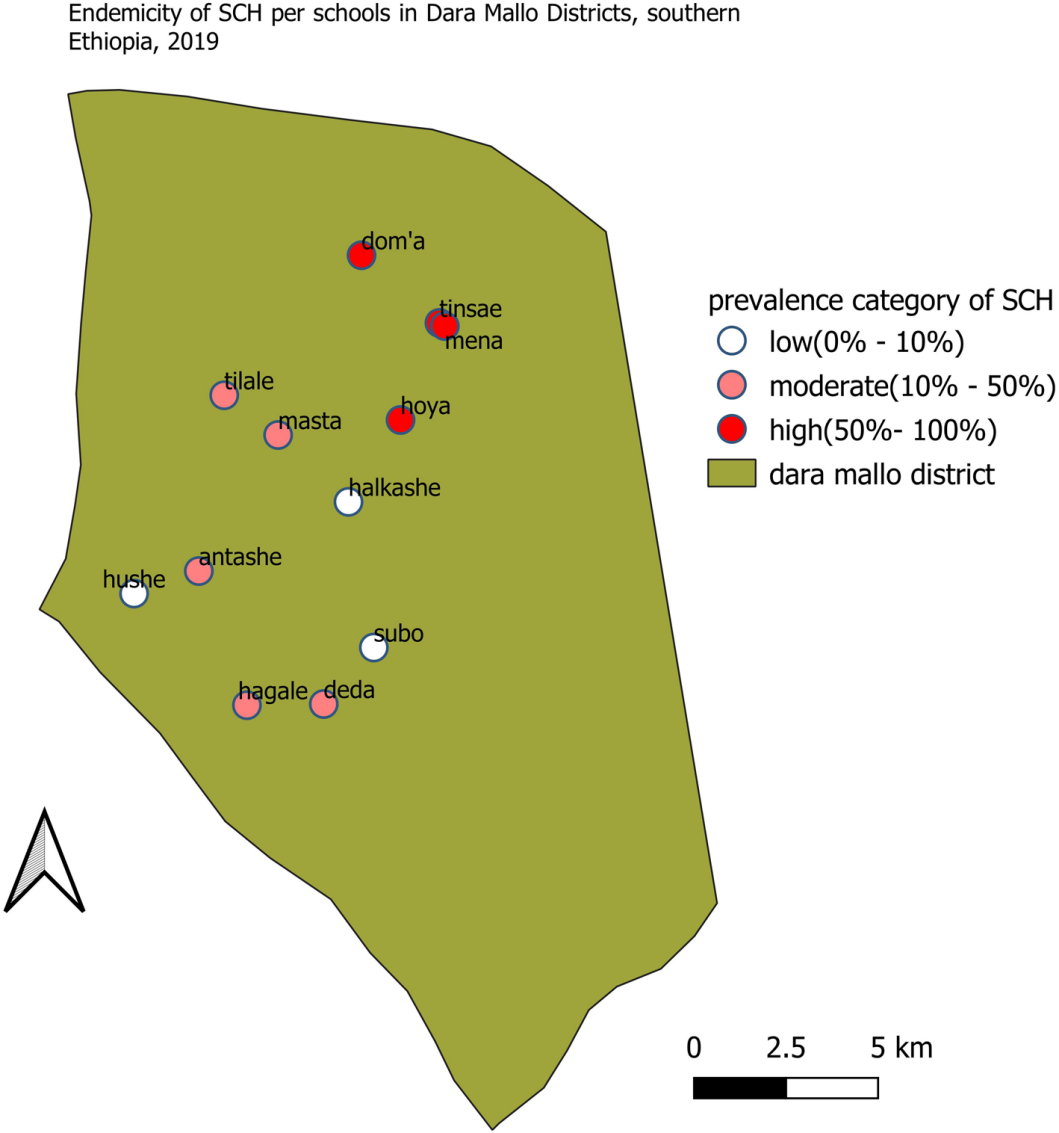
SCH infection endemicity per schools in Dara Mallo District in Southern Ethiopia, 2019.

The prevalence of *S. mansoni* infection also varied widely per school with the minimum prevalence observed was in Shella Subo primary school (3.2%) and the highest prevalence was in Hoya school (90.8%). Concerning the endemicity of infections per school involved in the study, only 3 out of 12 schools were low-endemic, 5 were moderate-endemic and 4 were high-endemic. The geospatial distribution of *S. mansoni* endemicity per school in the Dara Mallo district was shown in Fig. 3.

### Predictors of STH infection

After fitting the model in multivariable mixed-effects logistic regression, factors significantly influencing STH infections were district of the residence, gender of the SAC, occupational status of the household head, and wealth index of the household. Being resident in Dara Mallo district [AOR = 3.2; 95%CI 1.9–5.2]; male gender [AOR = 1.7; 95%CI 1.4–2.1], occupational status of the household head other than farmer/housewife [AOR = 0.5; 95%CI 0.3–0.8] and middle [AOR = 1.1; 95%CI 1.0–1.3] or high [AOR = 0.7; 95%CI 0.5–0.9] socioeconomic status (Table 3). Most of water access, sanitation, and hygiene-related factors significantly influenced STH infection in univariable mixed-effect logistic regression but they did not significantly predict STH infection by SAC in multivariable mixed-effect logistic regression (Table 4).

**Table 3:** Univariable and multivariable mixed effects logistic regression analysis of factors influencing STH infection among SAC in Dara Mallo and Uba Debretsehay districts, southern Ethiopia, 2019.

Factor	Categories	STH infected		COR (95%CI)	p-value	AOR (95%CI)	p-value
		No (%)	Yes (%)				
District**	Uba Debretsehay	1000 (73.4)	362 (26.6)	1	< 0.001	1	< 0.001
	Dara Mallo	389 (54.3)	328 (45.7)	2.8 (1.6–4.7)		3.2 (1.9–5.2)	
Place of residence*	Rural	1151 (64.7)	628 (35.3)	1		1	
	Semi-urban	122 (77.7)	35 (22.3)	0.4 (0.1–0.9)	0.036	0.5 (0.2–1.2)	0.137
	Urban	116 (81.1)	27 (18.9)	0.5 (0.1–1.6)	0.243	1.0 (0.3–2.8)	0.977
Residence house is	Private	1327 (66.4)	672 (33.6)	1	0.421		
	Not private	62 (77.5)	18 (22.5)	0.8 (0.4–1.4)			
Gender of SAC**	Female	748 (72.0)	291 (28.0)	1	< 0.001	1	< 0.001
	Male	641 (61.6)	399 (38.4)	1.7 (1.4–2.1)		1.7 (1.4–2.1)	
Age of SAC	7–9	1016 (66.5)	512 (33.5)	1	0.990		
	10–14	373 (67.7)	178 (32.3)	1.0 (0.8–1.3)			
Sex of household head	Male	1278 (66.2)	652 (33.8)	1	0.280		
	Female	111 (74.5)	38 (25.5)	0.8 (0.5–1.2)			
Occupation of the household head**	Farmer/housewife	1138 (64.0)	631 (36.0)	1	< 0.001	1	0.001
	Others	251 (83.1)	51 (16.9)	0.5 (0.3–0.7)		0.5 (0.3–0.8)	
Age of child mother	≤ 34	358 (69.6)	156 (30.4)	1			
	35–49	926 (66.2)	473 (33.8)	1.0 (0.8–1.3)	0.830		
	≥ 50	105 (63.3)	61 (36.7)	1.0 (0.7–1.5)	0.942		
Educational status of household head	Illiterate	754 (65.4)	399 (34.6)	1	0.064		
	Literate	635 (68.6)	291 (31.4)	0.8 (0.6–1.0)			
Occupation of SAC mother*	Housewife/farmer	1245 (65.6)	654 (34.4)	1	0.018		
	Others	144 (80.0)	36 (20.0)	0.6 (0.4–0.9)			
Age of SAC mother/caretaker	≤ 34	908 (68.0)	422 (32.0)	1			
	35–49	460 (65.0)	248 (35.0)	1.0 (0.8–1.3)	0.696		
	≥ 50	21 (60.0)	14 (40.0)	1.4 (0.6–2.9)	0.419		
Educational status of mother/caretaker*	Illiterate	976 (64.7)	533 (35.3)	1	0.013		
	Literate	413 (72.5)	157 (27.5)	0.7 (0.6–0.9)			
Education level of mother/ caretaker	< grade 7	221 (70.4)	93 (29.6)	1	0.531		
	≥ grade7	168 (77.1)	50 (22.9)	0.9 (0.5–1.4)			
Socioeconomic status of the household**	Low	412 (59.5)	281 (40.5)	1			
	Meddle	468 (67.3)	224 (32.3)	0.7 (0.6–0.9)	0.009	0.7 (0.6–1.0)	0.024
	High	508 (73.3)	185 (26.7)	0.6 (0.4–0.8)	< 0.001	0.7 (0.5–0.9)	0.004
Malnourished	No	1087 (67.2)	530 (32.8)	1	0.643		
	Yes	114 (61.6)	71 (38.4)	1.1 (0.8–1.5)			
Wasted (WAZ)	No	1138 (66.9)	563 (33.1)	1	0.658		
	Yes	66 (62.9)	39 (37.1)	1.0 (1.0–1.0)			
Under weighted (BAZ)*	No	1301 (66.5)	656 (33.5)	1	0.033	1	0.060
	Yes	82 (71.3)	33 (28.7)	1.1 (1.0–1.3)		1.1 (1.0–1.3)	
Stunted (HAZ)	No	1255 (67.1)	616 (32.9)	1	0.282		
	Yes	128 (63.7)	73 (36.3)	1.0 (1.0–1.1)			

*Significant only in univariate analysis. ** Significant in multivariable analysis.

**Table 4:** Univariable and multivariable mixed effects logistic regression analysis of sanitation and hygiene related factors influencing STH infection among SAC in Dara Mallo and Uba Debretsehay districts, southern Ethiopia, 2019.

Factor	Categories	STH infected		COR (95%CI)	p-value	AOR (95%CI)	p-value
		No (%)	Yes (%)				
Type of household latrine*	Had latrine	1312 (68.0)	617 (32.0)	1	0.025		
	Open field	77 (51.3)	73 (48.7)	1.6 (1.1–2.3)			
Toilet cleanliness condition*	Clean	333 (71.9)	130 (28.1)	1			
	Somewhat clean	819 (66.8)	405 (33.2)	1.4 (1.0–1.8)	0.034		
	Difficult to walk	237 (60.8)	153 (39.2)	1.5 (1.1–2.1)	0.020		
Drinking water source	Others	1109 (67.7)	530 (32.3)	1	0.571		
	Surface water	280 (63.6)	160 (36.4)	1.1 (0.8–1.7)			
Washed hand before last meal	Yes	1334 (67.4)	644 (32.6)	1	0.057		
	No/unknown	55 (54.5)	46 (45.5)	1.6 (1.0–2.6)			
Wash hand before meal with*	Not wash hand	55 (54.5)	46 (45.5)	1			
	Only water	992 (66.8)	492 (33.2)	0.6 (0.4–1.0)	0.075		
	Used soap/others	342 (69.2)	152 (30.8)	0.6 (0.3–0.9)	0.027		
Wash hand after toilet	Yes	1078 (66.6)	540 (33.4)	1	0.832		
	No/unknown	311 (67.5)	150 (32.5)	1.0 (0.8–1.4)			
Washed hand after toilet with	Not wash hand	311 (67.5)	150 (32.5)	1			
	Only water	720 (66.2)	368 (33.8)	1.0 (0.7–1.3)	0.913		
	Used soap/others	358 (67.5)	172 (32.5)	0.9 (0.7–1.3)	0.677		
Child walking on barefoot	No	907 (69.3)	401 (30.7)	1	0.284		
	Yes	482 (62.5)	289 (37.5)	1.1 (0.9–1.4)			
Child had shoe to wear	No	44 (50.6)	43 (49.4)	1	0.334		
	Yes	1345 (67.5)	645 (32.5)	0.8 (0.5–1.3)			
Worn shoe at the time of interview	No shoe to wear	44 (50.6)	43 (49.4)	1			
	No	100 (62.5)	60 (37.5)	0.8 (0.5–1.5)	0.557		
	Yes worn shoe	1245 (68.0)	587 (32.0)	0.8 (0.5–1.3)	0.324		
Nail of child is trimmed*	No	461 (63.7)	263 (36.3)	1	0.022		
	Yes	928 (68.5)	427 (31.5)	0.8 (0.6–1.0)			
Cleanliness of finger nail area*	Somewhat/clean	1178 (69.1)	527 (30.9)	1	0.050		
	Dirty	211 (56.4)	163 (43.6)	1.4 (1.0–1.8)			

*Significant only in univariate analysis.

## Discussion

The prevalence of STH infection, infection by *S. mansoni*, their intensity of infection, and infections associated risk factors among SAC after 5 years of MDA against STH in Dara Mallo and Uba Debretsehay districts were assessed in the present study. The prevalence of any STH infection was 33.3% and the overall prevalence of moderate-to-heavy STH infection among all children was 2.0%. The prevalence of *S. mansoni* infection and its heavy intensity of infection were 34.3% and 24.0% respectively. Infection by any STH among SAC in the study area was affected by the gender of the child, occupational status of the household head, and economic status, but none of the sanitation and hygiene-related factors influenced STH infection significantly.

The prevalence of any STH infection in Uba Debretsehay and Dara Mallo districts remain moderate endemic at the end of 2019 indicating achieving elimination of morbidity target which is defined as the prevalence of moderate-to-heavy for STH and heavy infections for *S. mansoni* < 1% among SAC^14^ and needs a continuation of the morbidity elimination strategies. The prevalence of STH infection in this study was in agreement with Chuahit district^17^ in Northern Ethiopia while it was higher than findings of similar studies undertaken in Jima town^18^ and Gurage Zone^19^ in Ethiopia. In contrast to this, the prevalence of STH infection among SAC in the study area was lower than that found in Hawassa town^20^ and west Shewa Zone^21^ in Ethiopia. The disagreements in the prevalence of STH infections in these studies could be related to the time of the data collection relative to the year when deworming has started, since deworming could reduce the worm burden and transmission. The high prevalence of STH infection in this study area as compared to other studies mentioned above might be related to the high prevalence of STH infection before deworming program was started in Ethiopia or the difference in the adherence of STH prevention measures. Except in Kenya^22,23^, the prevalence of STH infection was higher in the current study area than in other areas outside Ethiopia. It was higher than the prevalence of STH infection in Cameroon^24,25^ and Sudan^26^. The high prevalence observed in Kenya as compared to the current study might be due to differences in the data collection time relative to the last deworming or the frequency of deworming undertaken as a method used to eliminate STH infection associated morbidities. One of the two studies mentioned from Cameroon was undertaken 2 months post-MDA unlike the present study^24^.

Different factors influenced infection of STH in Uba Debretsehay and Dara Mallo districts. One of the socio-demographic factors that influenced STH infection by children was being male. Male children might be more engaged in playing with infested soil outside the houses than females that support their mother and other girls who were more concentrated in activities within the house compounds^23^. This was in agreement with findings from Gurage Zone^19^ and *T.trichuira* infection in Cameroon^25^. The other identified factor influencing STH infection was the economic level of the household. It is known that STH and other neglected tropical diseases were more common in economically disadvantaged populations. This was corroborated in the present study as those in middle or high economic status were at decreased risk of infection as compared to those in the lower economic category^1^. A community-based study carried out in northern Ethiopia also demonstrated that children from households with poor wealth Index were more likely to be infected by intestinal parasites^27^.

Poor access to improved water, sanitation, and hygiene (WASH) practice were one of the areas for intervention to eliminate STH and SCH. However, improved water source, hand washing after the last recent toilet and before last recent meals by child did not affect STH infection. One large-scale cluster-randomized trial was carried out to assess whether provision of water supply, hand washing facility, sanitation facility, drinking water, and provision of behavioral change education and promotion from 2014 to 2017 in Laos did not influence STH infection^28^. Similar to this study, a survey undertaken in Gurage Zone^19^ and in Jawi town^29^ revealed that STH infection was not affected by trimming nails. Another similar study undertaken in West Shewa Zone indicated that untrimmed nail and availability of latrine at household level did not associate with STH infection^21^. In contrast to the present study, other studies carried out in Ethiopia^17,29,30^ and abroad^22,23^ found a significant influence of WASH related factors on STH infections. The disagreement of these might be linked to social desirability bias as over-reporting of positive hygienic behavior than the real practice of the study participants^31^ and might be difference in the level of improved water access, sanitation and hygiene that can interrupt the transmission of the parasites.

The prevalence of *S. mansoni* in the Dara Mallo district was moderate-endemic though it was not targeted in the national MDA campaign for the last 5 years. This might be occurred due to a sampling problem during mapping as the sample collected from five schools^32^ would not show the full picture of the district. The transmission of *S. mansoni* was more focal and observed in schools that are in the Wacha cluster of the studied schools. This cluster involves schools in the town and those surrounding it. There were irrigation canals almost surrounding the town and which might be the major source for breading the snail hosts important to complete the life cycle of *S. mansoni*. The prevalence of *S. mansoni* infection in the present study was higher than other studies conducted in Ethiopia such as Jima town^33^, Manna^34^ and Chuahit^17^ district in northern Ethiopia, Sabatamit (rural Bahir dar)^35^ in Northern Ethiopia, in west Shewa Zone^21^ and Wondo district in west Arsi Zone^36^. The high prevalence of infection and intensity of infections were due to the absence of periodic MDA to reduce the transmission and morbidity of the infection in the present study area. *S. mansoni* infection was lower than the prevalence of its infection in Ljinga Island in Tanzania^37^ which could be related to the difference in the frequency of contact of children with the water bodies that inhabit *S. mansoni* infected snails. Children who had habit of swimming on water bodies were at higher risk of infection. In the majority of the studies, infection by SCH was positively affected by children who had the habit of swimming on water bodies: Chuahit^17^, Sabatamit rural Bahir Dar^35^, west Shewa zone^21^ and Korhogo in Côte d’Ivoire^38^.

There were limitations related to this study. One of these is due to the type of study design used. The cross-sectional study design, used in this study, is poor in generating evidence on the causal relationship. The strength of this study was using a large sample size to generate evidence on the infection status of children on STH and SCH and reaching children in remote and hard-to-reach areas.

## Conclusion

The prevalence of STH infection remained moderate in both districts with the highest prevalence observed in the Dara Mallo district. This implies that achieving the morbidity elimination target by 2020 is not achievable. Thus, stringent monitoring of the deworming process and coverage validation survey should be undertaken to improve the implementation of the interventions and accelerate the elimination of these diseases as a public health problem. More attention should be given to male children, children from low socioeconomic status and improving the level of access to improved water, sanitation and hygiene than the its mere presence. The clustered nature of *S. mansoni* infection in the Dara Mallo district pinpoints the importance of sub-district level mapping and initiating PC based on the mapping result. Furthermore, studies focusing on the transmission dynamics and interventions targeting the snail host shall be considered.

## Data Availability

The datasets used and/or analyzed during the current study will be available from the corresponding author on reasonable request.

## Abbreviations

AIC: Akaike Information Criterion
AOR: Adjusted Odds Ratio
CI: Confidence interval
COR: Crude odds ratio
GPS: Global Positioning System
IRB: Institutional Research Ethics Review Board
MDA: Mass Drug Administration
ODK: Open Data Kit
OR: Odds ratio
PC: Preventive chemotherapy
QGIS: Quantum Geographical Information System
SAC: School-aged children
SCH: Schistosomiasis
SD: Standard deviation
SNNPR: South Nations Nationalities and Peoples Region
SSA: Sub-Saharan Africa
STH: Soil-transmitted helminthiasis
WHO: World Health Organization
